# Provitamin A Carotenoids, Tocopherols, Ascorbic Acid and Minerals in Indigenous Leafy Vegetables from Tanzania

**DOI:** 10.3390/foods8010035

**Published:** 2019-01-19

**Authors:** Victoria Flavian Gowele, Joyce Kinabo, Theresia Jumbe, Carolyn Kirschmann, Jan Frank, Wolfgang Stuetz

**Affiliations:** 1 Department of Food Technology Nutrition and Consumer Sciences, College of Agriculture, Sokoine University of Agriculture, P.O. Box 3006, Morogoro, Tanzania; joyce_kinabo@yahoo.com (J.K.); tjumbe2000@yahoo.com (T.J.); 2 Institute of Nutritional Sciences, University of Hohenheim, Garbenstr. 28, 70599 Stuttgart, Germany; carolyn.kirschmann@gmx.net (C.K.); jan.frank@nutres.de (J.F.); wolfgang.stuetz@uni-hohenheim.de (W.S.)

**Keywords:** leafy vegetables, micronutrients, carotenoids, nutrient intake, Tanzania

## Abstract

The essential micronutrients in indigenous leafy vegetables (ILVs) could substantially contribute to the micronutrient supply in rural communities in Tanzania, but concentrations differ between species. Provitamin A carotenoids, tocopherols, ascorbic acid, minerals, and phytate were analysed in 13 different species using HPLC-, ICP-OES, and photometric techniques. Eight of the 13 ILVs, including *Amaranthus* ssp. and *Sesamum angustifolium*, had high β-carotene concentrations (2.91–4.84 mg/100 g fresh weight), which could provide ≥50% of vitamin A’s recommended nutrient intake (RNI). Six ILVs including *Cleome hirta* and *Sonchus luxurians* had high iron contents (34.5–60.4 mg/100 g, >50% RNI); *Amaranthus* ssp. represented the ILV with high calcium, magnesium and zinc contents (85%, 207% and 21% of RNI per 100 g); *Cleome hirta* and *Cleome gynandra* had high ascorbic acid contents (>15 mg/100 g, 34–35% of RNI), while *Sesamum angustifolium* was the only ILV with a high tocopherol content (7.34 mg α-TE/100 g). The highest phytate concentration was found in *Amaranthus* ssp., which could negatively affect its role as a very good source of minerals. Results indicate that the analysed ILVs could make a substantial contribution to the vitamin A and iron supply in the diets of rural Tanzanian populations.

## 1. Introduction

Indigenous leafy vegetables (ILVs) are acknowledged as part of a healthy diet due to their contribution to the dietary requirements of essential micronutrients such as vitamin A and C, iron, zinc, calcium, and magnesium [1,2,3]. Half of vitamin A and one-third of iron requirements in rural Tanzanian households are obtained through the consumption of indigenous leafy vegetables [4]. The typical daily main meal in Tanzania is made up of a cereal food such as rice or stiff porridge (ugali) made from maize or pearl millet flour, and is usually accompanied by a serving of cooked green leafy vegetables. These green leafy vegetables can be homegrown, bought at local markets, or harvested from the wild as indigenous species [4,5]. The term “indigenous” in this case refers to a crop species or variety genuinely native to a region, or to a crop introduced into a region where over a period of time it has evolved, although the species may not be native [4]. ILVs grow as weeds in the wild or in cultivated areas, but have also been domesticated through semi-cultivation or cultivation [6]. In addition, ILVs thrive with minimal care, are inexpensive, easily accessible, easy to cook, and could play an important role in ensuring micronutrient supply [3,7,8,9,10]. ILVs with high concentrations of provitamin A carotenoids and iron could essentially contribute in the reduction of vitamin A and iron deficiencies in rural areas where the diet is low in animal source foods [11]. The Tanzania Demographic and Health Survey in 2010 revealed a high prevalence of iron (>29%) and vitamin A deficiency (>35%) in women aged 15–49 years [12]. The present study is part of the ‘Scaling Up Nutrition’ project (www.scale-n.org) which focus on the development of nutrient-sensitive strategies to improve nutrition and health status of small-scale-farmer in Dodoma and Morogoro regions; in these areas ILVs are already part of the daily diet, but detailed data on the concentrations of individual provitamin A carotenoids, vitamins and minerals in the different species are inadequate. Therefore, thirteen species of ILVs were collected from the study areas in Chamwino and Kilosa districts in order to analyse the profile of micronutrients including provitamin A carotenoids, tocopherols, ascorbic acid, and the minerals iron, zinc, magnesium, and calcium. In addition, the concentration of phytate (phytic acid-inositol hexakisphosphate) as a naturally occurring anti-nutritional factor known to affect the bioavailability of minerals was determined [10].

## 2. Materials and Methods 

### 2.1. Description of the Study Area

The study was conducted in Dodoma and Morogoro regions in Tanzania. One district from each region was selected, Chamwino district in Dodoma and Kilosa district in Morogoro. Mzula and Chinoje villages in Chamwino and Tindiga and Mhenda villages in Kilosa district were selected based on factors such as geographical location, climate and consumption pattern of different indigenous vegetables (www.scale-n.org). Chamwino is located in the central plateau of Tanzania characterized by a dry Savannah and periodically semi-arid type of climate with long dry season starting late April to early December, and a short single wet season starting December to mid-April. The annual rainfall is between 350–500 mm. Kilosa District is located in eastern Tanzania characterized with sub-humid climatic condition with short rains starting in October to December and long rainfall period begin in February to May. This area has flat plains, highlands, and dry alluvial valleys with annual rainfall between 600–800 mm [13]. 

### 2.2. Sample Collection and Preparation

Thirteen different species of indigenous leafy vegetables were collected from their natural habitat during the wet season in April 2016 (Kilosa samples) and March 2017 (Chamwino samples), as shown in Table 1. Selection of the vegetable species for our research was based on the focus group discussion findings in the study areas where most women mentioned their wider consumption by the community. Identification of the vegetable samples was done by a botanist from the Department of Crop Science and Horticulture at Sokoine University of Agriculture (SUA). 

The leafy edible parts of the vegetables were separated from the main plant, washed with running tap water, air-dried at room temperature at the study sites, placed in black polyethylene bags, and subsequently transported to the laboratory at SUA. The first batch of samples from Kilosa were kept from April 2016 to August 2016 in a −30 °C freezer then transported on dry ice to the University of Hohenheim, Stuttgart in Germany; stored at −80 °C, freeze-dried, and analysed in September 2016. The second batch of samples from Chamwino were kept for two days in a −30 °C freezer, then immediately transported on dry ice to the University of Hohenheim, Stuttgart in Germany; stored at −80 °C for one week, then freeze-dried and analysed. The individual ILV species from both sample batches, were freeze-dried for 24 h (to constant weight), protected from light (using aluminium foil) using a Telstar freeze drier (2014 model LyoQuest; Telstar, Spain), ground in a mortar to a fine homogenous powder and stored in airtight containers protected from light; aliquots of the (freeze-dried) powder were finally weighed as samples for individual analyses on micronutrients and phytate contents. Details regarding botanical names, local names, villages of collection, and the conversion factors of the analysed ILVs are given in Table 1. Samples were weighed before and after freeze-drying and the respective factor for converting results per freeze-dried weight (FDW) to fresh weight (FW) was calculated according to the following Formula (1):

Conversion factor = (b − a)/(c − a)(1)

where a = weight of empty bottle; b = weight of the sample with the bottle before freeze-drying; and c = weight of the sample with the bottle after freeze-drying. Details of weight (or moisture) loss due to freeze-drying of the different ILV species are given in Table A1 (Appendix A).

### 2.3. Analytical Methods 

All determinations were done in freeze-dried samples (powder). Carotenoids, tocopherols, and ascorbic acid (AA) were analysed using High-Performance Liquid Chromatography (HPLC). The minerals iron, calcium, magnesium, zinc, and phosphorus were determined using Inductively Coupled Plasma-Optical Emission Spectrophotometry (ICP-OES), while phytic acid content was analysed after clean-up using a photometric method. Micronutrient and phytate contents in homogenized freeze-dried ILV samples were determined as milligrams per 100 g dry matter (mg/100 g) and finally converted and presented per fresh weight using the dry-to-fresh conversion factor (Table 1). The conversion from freeze-dried weight to fresh weight was calculated according to the following Formula (2):

Values per fresh weight = (Values per freeze − dried weight)/Conversion factor (2)

where (values per freeze − dried weight) are the concentrations per 100 g freeze-dried weight of respective micronutrients and phytic acid content measured by the HPLC, ICP-OES, and photometric method. Finally, all results (in the tables) are given in mg or µg/100 g of estimated fresh weight. 

#### 2.3.1. Determination of Carotenoids and Tocopherols

Carotenoids and tocopherols were extracted and analysed via HPLC, as previously described by Stuetz et al. [14]. Briefly, 100 mg of freeze-dried ILV samples were saponified in screw-capped glass tubes under stirring in a light protected water bath (2.5 h, at 38 °C) by adding 500 µL of KOH (50% *w*/*w*), and 1 mL ethanol containing β-apo-8′-carotenal methyloxime (1 µL/mL) as an internal standard. After saponification, 2 mL of saline solution (15% *w*/*V*) was added, then samples were neutralised with 500 µL of glacial acetic acid. The fat-soluble components were extracted with hexane (2 × 1 mL), and combined fractions were evaporated in a rotational vacuum evaporator (RVC 2-33 IR, Christ, Osterode am Harz, Germany). The residue was redissolved in 70 µL ethanol (>96%) and 210 µL acetonitrile in order to be analyzed using RP-HPLC, UV-vis (450 nm for carotenoids) and fluorescence detection (Excitation set at 298 nm and Emission set at 328 nm for α-/γ-tocopherol). All reagents and solvents were of analytical and (ultra) gradient HPLC grade. N-hexane, 1, 4-dioxane, potassium hydroxide solution (50%), acetic acid (100%), and ethanol were from Carl Roth GmbH + Co. KG (Karlsruhe, Germany), while methanol and acetonitrile were from J.T. Baker (Deventer, The Netherlands).

Recommended daily safe intake of 500 µg retinol equivalents (RE) for vitamin A intake and daily acceptable intakes of 7.5 mg of α-tocopherol equivalents (α-TE) for vitamin E intake among females aged 19–50 years were used to estimate the contribution of ILVs [15]. Conversion factors of 12:1 for β-carotene and 24:1 for other β-cryptoxanthin and α-carotene were applied for the calculation of RE, while α-TE were calculated using conversion factors of 1:1 for α-tocopherol and 10:1 for γ-tocopherol [15].

#### 2.3.2. Determination of Ascorbic Acid (AA)

Ascorbic acid in 25 mg of freeze-dried samples were extracted with a mixture of 550 µL of freshly prepared 10% (*w*/*w*) meta-phosphoric acid (MPA) and 50 µL of 20% (*w*/*w*) tris-(2-carboxyethyl)-phosphine (TCEP, C_9_H_15_O_6_P·HCl) solution as the reduction agent; samples were incubated for 5 min on ice (in the dark, protected from light), then vortex-mixed for 3 min, and centrifuged at 17,000 × *g* for 10 min; 20 µL of supernatants were analysed on AA content using a Shimadzu Prominence HPLC, a Reprosil-Pur 120 C18 AQ analytical column (5 µm, 250 × 4.6 mm, Dr. Maisch GmbH, Ammerbuch, Germany), sodium dihydrogen-phosphate buffer (0.1 M NaH_2_PO_4_ x 3H_2_O, set to pH 2.5) as a mobile phase at a flow rate of 1 mL/min and an UV-Vis detector (SPD-20A, Shimadzu, Kyoto, Japan) set at 245 nm [16]. TCEP, MPA, and reagents were from Carl Roth GmbH + Co. KG, Karlsruhe, Germany. The recommended nutrient intake (RNI) was calculated based on 45 mg/day recommendation of Vitamin C for women 19–50 years [15].

#### 2.3.3. Determination of Minerals 

Iron (Fe), zinc (Zn), calcium (Ca), and magnesium (Mg) contents in 250 mg of samples were determined by ICP-OES after microwave-heated nitric acid digestion using an ultraclave, as previously described [17]. The RNI were calculated based on, iron (58.8 mg/day, 5% bioavailability); zinc (9.8 mg/day, low bioavailability); calcium (1000 mg/day) and magnesium (220 mg/day) for women 19–50 years [15].

#### 2.3.4. Determination of Phytate (and Its Ratio to Analysed Minerals)

Phytate (inositol hexakisphosphate, IP6) was quantitatively measured by photometry [18] after extraction and clean up using solid-phase extraction (SPE) and anion-exchange purification [19]. In brief, 150 mg of samples were extracted using 0.1 M HCl (1.5 mL) and sonication for 30 min. Following samples were centrifuged twice (60 min at 13,300 rpm) and clear supernatants of ILV extracts (500 µL) were diluted (1:20) and adjusted to pH 6 in order to be transferred to SPE glass cartridges filled with 500 mg of anion exchange resin (AG1-X8 resin, Bio-Rad laboratories, Inc., Hercules, CA, USA). Samples were washed (0.1 M NaCl to remove free phosphate) and phytates were eluted with 0.7 M NaCl. Samples were adjusted to pH 3, mixed with Wade reagent (0.06% FeCl_3_∙6H_2_O + 0.6% sulfosalicylic acid) and absorbance was measured at 490 nm. Phytate (phytic acid) standards in concentrations from 25 µg/mL to 200 µg/mL and blanks (Wade reagent) were used for calibration. To predict the bioavailability of calcium, iron and zinc; molar weights (phytate: 660 g/mol; Ca: 40 g/mol; Fe: 56 g/mol; and Zn: 65 g/mol) were used to calculate the (molar) ratio of phytate to individual mineral concentrations of the ILVs [20].

### 2.4. Statistical Analysis

Results are concentrations per wet weight (fresh weight) and described as means and standard deviations. Data used for provitamin A carotenoids, ascorbic acid and tocopherols were means of triplicate (*n* = 3) determinations while for minerals and phytate were of duplicate (*n* = 2) determinations. Multiple comparisons between ILVs were performed by one-way analysis of variance (ANOVA) and post-hoc Tukey’s HSD (Honestly Significant Difference) test; statistical significance was considered at a *p* value < 0.05. All statistical analyses were carried out using IBM SPSS software (Version 23, IBM Corp., Armonk, NY, USA). 

## 3. Results and Discussion

### 3.1. Provitamin A Carotenoids Contents

The provitamin A carotenoids contents and the contribution of different vegetable species to the RNI of vitamin A (retinol equivalents-RE) are presented in Table 2. Table 2 shows a range of β-carotene contents between 1.01 and 4.84 mg/100 g. *Amaranthus* spp. (4.84 mg/100 g) had a high β-carotene content compared to other ILVs. *Sesamum angustifolium* (4.06 mg/100 g), *Corchorus trilocularis* (4.04 mg/100 g), and *Justicia heterocarpa* (3.84 mg/100 g) were also rich in β-carotene (Table 2). In comparison to other analysed ILVs, *Sesamum angustifolium* (0.18 mg/100 g) and *Justicia heterocarpa* (0.95 mg/100 g) had the highest content of β-cryptoxanthin and α-carotene, respectively.

β-Carotene contents of *Cleome gynandra*, *Sonchus luxurians,* and *Amaranthus* spp., from this study, were in agreement with other reported values of ILVs in Tanzania [4,21]. Limited results are available for β-cryptoxanthin and α-carotene comparison. A study on ILVs from Cameroon [22], reported contents (per dry weight) of β-cryptoxanthin (0.21 ± 0.03 mg/100 g) and α-carotene (0.21 ± 0.05 mg/100 g) in *Ceratotheca sesamoides*. However, a strong comparison cannot be made between the reported contents and data from the present study, as different drying techniques were used: Sun drying in the previous work versus freeze-drying in the present study. 

The stability and retention of carotenoids in food are influenced by various factors including their chemical nature, storage time, storage conditions, cooking, and preparation methods [2,23]. Indigenous leafy vegetables in Tanzania are mostly prepared by steaming, boiling, or stir-frying together with other vegetables such as tomatoes and onions. Steaming and stir-frying with oil are mentioned as desirable methods for preparing ILVs in view of carotenoids retention and bioavailability [2,23,24]. Due to different plant matrices, the bioavailability of carotenoids from orange-pigmented fruits was superior to that of dark-green leafy vegetables, as reported by De Pee et al. [25]. However, the bioavailability of β-carotene and other carotenoids can be enhanced through food processing (cooking, homogenisation) and fat in the diet [26,27,28]. Moderate cooking and/or mechanical homogenisation, which destroys the cell walls, increase the release of carotenoids, while a sufficient amount of dietary fat (3–5 g per meal), especially if rich in unsaturated fatty acids, is essential for maximal carotenoid absorption from vegetables [27,28]. Results in the present study indicate *Amaranthus* spp., *Justicia heterocarpa*, *Sesamum angustifolium*, *Corchorus trilocularis*, *Amaranthus spinosus*, *Ipomoea pandurata*, *Vigna unguiculata* and *Cleome gynandra* as rich sources of vitamin A since the consumption of 100 g of these vegetable species can provide between 50% to 84% of the RNI for retinol equivalents (RE). A study conducted in central and North-Eastern parts of Tanzania [29] reported an average per capita vegetable intake of 207 g per day, suggesting a contribution of 104 to 174 percent of the RNI for retinol equivalents (RE), and thus underlining an important contribution of these ILVs to ensuring vitamin A intake by the population in the study areas. 

### 3.2. Tocopherols and Ascorbic Acid Contents

Low contents of γ- and α-tocopherol were observed in almost all analysed samples; *Sesamum angustifolium* being the exception with a high concentration of α-tocopherol (7.29 mg/100 g) (Table 3). In comparison to other ILVs, *Amaranthus* spp. exhibited the highest γ-tocopherol content (0.67 mg/100 g) followed by *Sesamum angustifolium* (0.54 mg/100 g). A 100 g portion of *Sesamum angustifolium* with the highest level of α-tocopherol equivalents (α-TEs) could provide 98% of the acceptable intake for vitamin E (Table 3). This could be explained by the presence of immature seeds that were part of the leaves in the sample; further research and analysis on separated leaves and seeds are required to describe and confirm *Sesamum angustifolium* as an excellent source for vitamin E. Until now, little information is available on the tocopherol contents of *Sesamum angustifolium* leaves. One previous study on *Sesamum angustifolium* seeds [30], reported total tocopherol contents of approximately 760 mg/kg oil, consisting γ- and δ-tocopherol, but no α-tocopherol. Studies done on the leaf of the sesame species *Sesamum indicum* reported the presence of α-tocopherol content (0.55 mg/100 g edible portion) but no detection of γ-tocopherol [31]. Vitamin E is the major lipid-soluble antioxidant that provides protection against lipid peroxidation and protects components of cell membranes from oxidation by free radicals, thus preventing or delaying the onset of chronic diseases associated with reactive oxygen species molecules [15,32]. The richest sources of tocopherols include nuts, seeds, and vegetable oils [32]. Results from the present study suggest further research into the contribution of *Sesamum angustifolium* as a source of tocopherols in the diet.

The ascorbic acid contents of 13 analysed ILVs ranged between 0.08 mg/100 g for *Justicia heterocarpa*, to 15.6 mg/100 g for *Cleome hirta* (Table 3). In previous studies a vitamin C content of 2 mg/100 g in *Amaranthus* spp., 9 mg/100 g in *Vigna unguiculata* and 2 mg/100 g in *Cleome gynandra* [3] was reported, which by contrast shows higher contents for *Amaranthus* spp and *Vigna unguiculata* but lower for *Cleome gynandra* (Table 3). Ascorbic acid values for *Cleome gynandra* are in agreement with that reported in other studies [1,33]. Variations in the contents of ascorbic acid in ILVs are known to be related to various factors such as plant variety or species, maturity stage, storage time and high temperature [33,34]. Vitamin C is a water-soluble antioxidant required for normal metabolic functions of the body and plays an important role as an enhancer of dietary Fe absorption [35,36]. Since non-heme Fe is the only Fe compound in plants, its absorption is said to be improved by dietary constituents such as ascorbic acid and other organic acids [37]. Five of the analysed ILVs could contribute more than 10% of the RNI by 100 g fresh weight, with *Cleome hirta* and *Cleome gynandra* reaching 35% and 34% of the RNI, and thus the reported average per capita vegetable intake of 207 g per day [29], would provide between 71 and 72 percent of the RNI for vitamin C (Table 3). Since longer storage times and higher temperatures reduce the ascorbic acid contents, a significant ascorbic acid supply through *Cleome hirta* and *Cleome gynandra* can only be achieved when these leaves are utilized immediately after harvesting. Additionally, shorter cooking times, with low water content, as well as the consumption of the cooking water are also recommended as measures to ensure ascorbic acid retention and intake [38].

### 3.3. Mineral Contents

The mean values of the vegetable mineral contents and their contribution to the RNI are presented in Table 4 and Table 5. Considerable amounts of Fe (2.4–60.4 mg/100 g) Zn (0.37–2.05 mg/100 g), Ca (100–853.7 mg/100 g), and Mg (37.7–455.8 mg/100 g) were found in the analysed ILV species. The ILVs with highest Fe contents were *Amaranthus* spp. (60.4 mg/100 g), *Cleome hirta* (56.4 mg/100 g), *Corchorus trilocularis* (43.5 mg/100 g), *Sonchus luxurians* (42.2 mg/100 g), *Cleome gynandra* (39.0 mg/100 g), and *Ceratotheca sesamoides* (34.5 mg/100 g). *Amaranthus* spp. also contained the highest amount of Zn (2.05 mg/100 g) in comparison to other ILVs (Table 4). Previous studies in Tanzania [4,39] reported Fe and Zn contents in *Amaranthus* spp., *Bidens pilosa*, *Sonchus luxurians*, *Chorchorus trilocularis,* and *Vigna unguiculata* in agreement with the ones in the present study. In a review on *Bidens pilosa* [2], Fe contents ranging between 2.0 to 6.0 mg/100 g fresh weight, Zn between 0.9 to 2.6 mg/100 g fresh weight, and Ca between 162 to 340 mg/100 g fresh weight were similar with contents reported in the present study. Other researchers [1,40,41], however, reported lower Fe concentrations (2.48–14.4 mg/100) in *Bidens pilosa*, *Cleome gynandra*, *Amaranthus spinosus,* and *Corchorus trilocularis* compared to those reported in the present study. The Zn content for *Bidens pilosa* was in agreement with the one reported by Reference [41]. 

Iron plays numerous biochemical roles in the body including the formation of red blood cells [9]. Limited supply of Fe in most usual African diets is due to low intake of animal-based foods [42]. ILVs such as *Amaranthus* spp., *Cleome hirta*, *Corchorus trilocularis* and *Sonchus luxurians* with high Fe content which could contribute 103%, 96%, 74%, and 72% to the RNI per 100 g, respectively, can be recommended to alleviate iron deficiency anaemia in communities with limited access to animal food sources. 

Zinc is important in gastrointestinal and immune functions, energy metabolism and as a co-enzyme in numerous biochemical reactions in the body [2,9]. Low contents of Zn were recorded in most of the analysed ILVs (Table 4). In comparison to other analysed ILV species, *Amaranthus* spp. had a high Zn content (2.05 mg/100 g), which could contribute to 21% to the RNI (Table 4). Similar results were reported in studies conducted in South Africa on *Amaranthus spinosus*, *Bidens pilosa*, *Cleome gynandra*, and *Vigna unguiculata* [3,41]. Overall, these results show that the analysed ILVs can contribute to a lesser extent to Zn nutrition in comparison to Fe. 

The range of Ca in the ILVs was between 100 mg/100 g in *Ipomoea obscura* and 853.7 mg/100 g *Amaranthus* spp., while for Mg it was between 37.7 mg/100 g in *Ipomoea obscura* and 455.8 mg/100 g in *Amaranthus* spp. (Table 5). The Ca content was higher in *Amaranthus* spp., followed by *Justicia heterocarpa*: an estimated consumption of 100 g could contribute 85% (*Amaranthus* spp.) and 48% (*Justicia heterocarpa*) of the RNI for Ca. In comparison to other analysed ILVs, the Mg content in *Amaranthus* spp. (455.8 mg/100 g), and *Amaranthus spinosus* (202.9 mg/100 g) was high, contributing a possible 207% and 92% of the RNI, respectively. Ca contents of *Bidens pilosa* and *Cleome gynandra* were in similar ranges of those reported by [3,41]. The values of magnesium in *Amaranthus spinosus* and *Cleome gynandra* were in agreement with the ones reported by [41]. Ca and Mg play important roles in development and maintenance of healthy bones and muscles [9]. A 100 g portion of the analysed ILVs did not meet the RNI for calcium but *Amaranthus* spp. and *Justicia heterocarpa* can be regarded as valuable Ca sources with possible contributions of 85% and 48% to the RNI (Table 5). *Amaranthus* spp. was the ILV with a high Mg content, reaching 2-fold the RNI per 100 g fresh weight. The bioavailability of Ca and Mg is dependent on the age and sex of an individual, fat content in the diet and the presence of antinutrients [2]. Therefore, *Amaranthus* spp., *Amaranthus spinosus,* and *Ceratotheca sesamoides* could potentially contribute towards the dietary requirements of these two minerals when cooking methods such as boiling, steaming, and stir-frying with oil are applied [7,9,24,43].

The quantitative analysis on minerals in ILVs revealed considerable high contents of Fe, Ca, and Mg in certain ILV species and therefore an important contribution to recommended daily mineral intakes. Most notable are the high concentrations of Fe, Ca and Mg in *Amaranthus* spp. compared to other ILVs. *Cleome gynandra* and *Cleome hirta* could serve as important ILVs in iron nutrition due to their appreciable contents of ascorbic acid that enhances Fe absorption. By contrast, the analysed ILVs could not serve as very good sources of Zn, since only 21% of the RNI was reached by the highest Zn content in *Amaranthus* spp. (Table 5). However, in comparison to the Zn contents of boiled rice (0.5 mg/100 g) and maize stiff porridge (0.6 mg/100 g), which are important staple dishes in Tanzania [44], *Amaranthus* spp. has more zinc content and this suggests that its integration could improve Zn content in the consumed diets.

### 3.4. Phytate Content

The phytate contents and molar ratios along with the suggested critical values for estimating the effect of phytate on the bioavailability of Fe, Zn, and Ca are presented in Table 6. *Amaranthus* spp. (739 mg/100 g) and *Amaranthus spinosus* (334 mg/100 g) had the highest phytate contents compared to other ILVs (Table 6). The calculated phytate: Iron molar ratios for six ILV samples (Table 6) were above the suggested critical level (>1) indicating poor iron bioavailability [45,46]. In this context, *Cleome gynandra*, *Cleome hirta*, *Corchorus trilocularis*, *Sonchus luxurians,* and *Ceratotheca sesamoides* with phytate:iron molar ratio <l are better sources of bioavailable iron (Table 6). The phytate: zinc molar ratios of *Ceratotheca sesamoides*, *Sonchus luxurians*, *Bidens pilosa*, *Cleome hirta* and *Justicia heterocarpa* were below the suggested critical level of 15, above which zinc bioavailability is seriously impaired [46]. *Amaranthus* spp. had high contents of Ca, Fe, and Zn (Table 4, Table 5 and Table 6), a low phytate:calcium (0.05), a still acceptable phytate:iron (1.04), but an unfortunately high phytate:zinc molar ratio (35.71). However, in consideration of the applied RNI for low zinc and 5% iron bioavailability, *Amaranthus* spp. can serve as an important source of Fe and Zn in the diet. The phytate: Calcium molar ratios in all the leafy vegetable samples ranged between 0.01–0.07, which is well below the critical level of 0.24, above which it is said that calcium bioavailability is impaired [46]. This suggests that the phytate contents of the analysed ILVs will not have a substantial impact on the calcium bioavailability. 

Phytate, which is the major storage form of phosphorus in plants [2] has the ability to form chelates with di-and tri-valent metallic ions such as Zn, Fe, Mg, and Ca to form poorly soluble compounds that are not readily absorbed from the gastrointestinal tract, thus decreasing their bioavailability [47]. Previous studies reported that traditional food preparation and cooking methods (fermentation, boiling, or frying) can significantly reduce phytate content in vegetables [7,9,10,43]. Therefore, the diminishing effect on phytate content by suggested cooking methods can even increase the contribution of ILVs as important sources of Fe, Zn, and Ca. 

## 4. Conclusions

The analysed indigenous leafy vegetables (ILVs) contain substantial amount of provitamin A carotenoids, minerals, but also the anti-nutritional factor phytate. There are differences in concentrations of essential micronutrients between ILV species and thus diverse consumption is beneficial in terms of micronutrient supply. The remarkable contents of iron and β-carotene revealed in the ILVs can serve an important role to safeguard iron and vitamin A supply in the study area. In rural settings where households rely on the consumption of ILVs improved preparation and processing methods to ensure carotenoids retention and reduction of phytate would be further measures to ensure vitamin A and iron supply in the population.

## Figures and Tables

**Table 1:** Vegetable samples origin and their conversion factors.

Sample Origin-District	Botanical Name	Local Names	Sample Origin-Village	Conversion Factor *
Chamwino	*Ceratotheca sesamoides*	Ilende	Chinoje	4.34
	*Vigna unguiculata*	Safwe	Chinoje	5.73
	*Cleome gynandra*	Mzimwe	Mzula	6.97
	*Cleome hirta*	Mhilile	Mzula	7.46
	*Ipomoea obscura*	Sagula sagula	Mzula	11.55
	*Ipomoea pandurata*	Chiwandagulu	Mzula	8.54
Kilosa	*Bidens pilosa*	Mashona nguo	Mhenda	6.42
	*Justicia heterocarpa*	Mwidu	Mhenda	5.22
	*Sesamum angustifolium*	Mlenda ufuta	Mhenda	3.99
	*Amaranthus spinosus*	Mchicha chawa	Tindiga	6.56
	*Amaranthus* spp.	Mchicha bwasi	Tindiga	2.42
	*Corchorus trilocularis*	Mlenda mgunda	Tindiga	5.65
	*Sonchus luxurians*	Sunga	Tindiga	8.19

* Conversion factor = FW/FDW, fresh weight/freeze-dried weight.

**Table 2:** Provitamin A carotenoids contents (mg/100 g fresh weight) of ILVs *.

Botanical Names	β-Cryptoxanthin	α-Carotene	β-Carotene	RE ^1^ (µg/100 g)	% of RNI ^2^
*Amaranthus spinosus*	0.04 ± 0.01 ^f,g^	0.26 ± 0.01 ^b,c^	3.65 ± 0.17 ^a,b,c^	317 ± 14 ^a,b,c^	63
*Amaranthus* spp.	0.13 ± 0.01 ^b,c,d^	0.32 ± 0.01 ^b^	4.84 ± 0.16 ^a^	422 ± 13 ^a^	84
*Bidens pilosa*	0.05 ± 0.01 ^f,g^	0.07 ± 0.01 ^c,d^	2.07 ± 0.07 ^c,d,e^	178 ± 6 ^c,d,e^	36
*Cleome gynandra*	0.13 ± 0.02 ^b,c,d^	0.05 ± 0.02 ^d^	2.91 ± 0.41 ^b,c,d^	250 ± 36 ^b,c,d^	50
*Cleome hirta*	0.06 ± 0.03 ^e,f,g^	0.01 ± 0.01 ^d^	2.75 ± 0.20 ^b,c,d^	232 ± 17 ^b,c,d^	46
*Corchorus trilocularis*	0.11 ± 0.01 ^c,d,e^	0.09 ± 0.01 ^c,d^	4.04 ± 0.57 ^a,b^	345 ± 48 ^a,b^	69
*Ceratotheca sesamoides*	0.14 ± 0.01 ^b,c^	0.07 ± 0.02 ^c,d^	1.96 ± 0.35 ^d,e^	172 ± 30 ^d,e^	34
*Ipomoea obscura*	0.09 ± 0.01 ^c,d,e,f^	0.07 ± 0.01 ^c,d^	1.01 ± 0.04 ^e^	90 ± 3 ^e^	18
*Ipomoea pandurata*	0.05 ± 0.03 ^f,g^	0.16 ± 0.01 ^b,c,d^	2.99 ± 0.15 ^b,c,d^	258 ± 13 ^b,c,d^	52
*Justicia heterocarpa*	0.07 ± 0.01 ^e,f,g^	0.95 ± 0.27 ^a^	3.84 ± 1.45 ^a,b^	363 ± 132 ^a,b^	73
*Sesamum angustifolium*	0.18 ± 0.01 ^a,b^	0.06 ± 0.01 ^c,d^	4.06 ± 0.39 ^a,b^	349 ± 33 ^a,b^	70
*Sonchus luxurians*	0.03 ± 0.01 ^g^	0.03 ± 0.01 ^d^	2.15 ± 0.03 ^c,d,e^	181± 2 ^c,d,e^	36
*Vigna unguiculata*	0.08 ± 0.01 ^d,e,f,g^	0.12 ± 0.05 ^b,c,d^	2.96 ± 1.26 ^b,c,d^	255 ± 107 ^b,c,d^	51

* ILVs, indigenous leafy vegetables: values are means ± SD; values within a column not sharing a common superscript letter (a,b,c,d,e,f,g) are significantly different (Tukey ≤ 0.05). ^1^ RE, retinol equivalents (sum of RE), for which 1 RE = 12 μg β-carotene or 24 μg α-carotene or 24 μg β-cryptoxanthin. ^2^ RNI, recommended nutrient intakes by WHO/FAO [15]; values as (microgram) for female adults (19–50 years).

**Table 3:** Tocopherols and ascorbic acid contents (mg/100 g fresh weight) in ILVs *.

Botanical Names	γ-Tocopherol	α-Tocopherol	α-TE ^1^	% AI ^2^	Ascorbic Acid	% RNI ^3^
*Amaranthus spinosus*	0.01 ± 0.01 ^c^	0.01 ± 0.01 ^b^	0.01 ± 0.01 ^b^	0	0.30 ± 0.02 ^g^	1
*Amaranthus* spp.	0.67 ± 0.05 ^a^	0.01 ± 0.01 ^b^	0.07± 0.01 ^b^	1	0.48 ± 0.01 ^f,g^	1
*Bidens pilosa*	0.02 ± 0.01 ^c^	0.03 ± 0.02 ^b^	0.03 ± 0.02 ^b^	0	1.20 ± 0.05 ^f,g^	3
*Cleome gynandra*	0.01 ± 0.01 ^c^	0.02 ± 0.01 ^b^	0.02 ± 0.01 ^b^	0	15.44 ± 0.91^a^	34
*Cleome hirta*	0.01 ± 0.01 ^c^	0.01 ± 0.01 ^b^	0.01 ± 0.01 ^b^	0	15.60 ± 0.24^a^	35
*Corchorus trilocularis*	0.04 ± 0.06 ^c^	0.04 ± 0.03 ^b^	0.04 ± 0.04 ^b^	1	0.25 ± 0.01^g^	1
*Ceratotheca sesamoides*	0.02 ± 0.01 ^c^	0.03 ± 0.01 ^b^	0.03 ± 0.01 ^b^	0	6.48 ± 0.07^d^	14
*Ipomoea obscura*	0.01 ± 0.01 ^c^	0.01 ± 0.01 ^b^	0.01 ± 0.01 ^b^	0	1.60 ± 0.15 ^f^	4
*Ipomoea pandurata*	0.04 ± 0.01 ^c^	0.07 ± 0.01 ^b^	0.07 ± 0.01^b^	1	11.28 ± 0.13 ^b^	25
*Justicia heterocarpa*	0.03 ± 0.01^c^	0.02 ± 0.01 ^b^	0.02 ± 0.01 ^b^	0	0.08 ± 0.01^g^	0
*Sesamum angustifolium*	0.54 ± 0.06 ^b^	7.29 ± 0.75 ^a^	7.34 ± 0.75 ^a^	98	4.09 ± 0.22 ^e^	9
*Sonchus luxurians*	0.01 ± 0.01 ^c^	0.01 ± 0.01 ^b^	0.01± 0.01 ^b^	0	0.68 ± 0.03 ^f,g^	2
*Vigna unguiculata*	0.04 ± 0.01 ^c^	0.02 ± 0.01 ^b^	0.02 ± 0.01 ^b^	0	8.96 ± 0.01 ^c^	20

* ILVs, indigenous leafy vegetables: values are means ± SD; values within a column not sharing a common superscript letter (a,b,c,d,e,f,g) are significantly different (Tukey ≤ 0.05). ^1^ α-TEs, α-tocopherol equivalents (1 mg α-TE = 1 mg α-tocopherol or 10 mg γ-tocopherol). ^2^ AI, acceptable intake [15]. ^3^ RNI, recommended nutrient intakes by WHO/FAO [15]; values in (milligram) for female adults (19–50 years).

**Table 4:** Iron and zinc contents (mg/100 g fresh weight) of ILVs *.

Botanical Names	Iron	% of RNI ^1^	Zinc	% of RNI ^1^
*Amaranthus spinosus*	19.8 ± 1.7 ^d^	34	0.82 ± 0.01 ^b^	8
*Amaranthus* spp.	60.4 ± 3.6 ^a^	103	2.05 ± 0.01 ^a^	21
*Bidens pilosa*	3.3 ± 0.2 ^g^	6	0.86 ± 0.01 ^b^	9
*Cleome gynandra*	39.0 ± 2.7 ^b^^,^^c^	66	0.46 ± 0.01 ^e^	5
*Cleome hirta*	56.4 ± 2.5 ^a^	96	0.46 ± 0.01 ^e^	5
*Corchorus trilocularis*	43.5 ± 1.1^b^	74	0.46 ± 0.01^e^	5
*Ceratotheca sesamoides*	34.5 ± 0.7 ^c^	59	0.67 ± 0.01 ^d^	7
*Ipomoea obscura*	19.6 ± 0.4 ^d^	33	0.38 ± 0.01^f^	4
*Ipomoea pandurata*	10.3 ± 0.0 ^e,f^	18	0.37 ± 0.02 ^f^	4
*Justicia heterocarpa*	10.5 ± 0.5 ^e,f^	18	0.75 ± 0.04 ^c^	8
*Sesamum angustifolium*	2.4 ± 0.3 ^g^	4	0.72 ± 0.01 ^c^	7
*Sonchus luxurians*	42.2 ± 0.7 ^b^	72	0.40 ± 0.01 ^f^	4
*Vigna unguiculata*	5.1 ± 0.1 ^f,g^	9	0.41 ± 0.01 ^f^	4

* ILVs, indigenous leafy vegetables: values are means ± SD; values within a column not sharing a common superscript letter (a,b,c,d,e,f,g) are significantly different (Tukey ≤ 0.05). ^1^ RNI, recommended nutrient intakes by WHO/FAO [15]; values as (milligram) for female adults (19–50 years).

**Table 5:** Calcium and magnesium (mg/100 g fresh weight) of ILVs *.

Botanical Names	Calcium	% of RNI ^1^	Magnesium	% of RNI ^1^
*Amaranthus spinosus*	289.1 ± 9.2 ^c,d^	29	202.9 ± 0.3 ^b^	92
*Amaranthus* spp.	853.7 ± 24.9 ^a^	85	455.8 ± 0.1 ^a^	207
*Bidens pilosa*	163.1 ± 1.1 ^g,h^	16	60.0 ± 0.2 ^i^	27
*Cleome gynandra*	260.1 ± 2.8 ^d,e^	26	70.1 ± 0.1 ^fg^	32
*Cleome hirta*	310.5 ± 1.3 ^c^	31	44.8 ± 0.0 ^j^	20
*Corchorus trilocularis*	167.3 ± 2.9 ^g^	17	62.0 ± 0.4 ^i^	28
*Ceratotheca sesamoides*	248.8 ± 3.1 ^e,f^	25	129.4 ± 1.8 ^c^	59
*Ipomoea obscura*	100.0 ± 1.7 ^i^	10	37.7 ± 0.5 ^l^	17
*Ipomoea pandurata*	143.3 ± 1.0 ^g,h^	14	67.2 ± 0.6 ^h^	31
*Justicia heterocarpa*	478.6 ± 3.3 ^b^	48	79.7 ± 0.1 ^e^	36
*Sesamum angustifolium*	168.4 ± 5.1 ^g^	17	83.7 ± 1.4 ^d^	38
*Sonchus luxurians*	135.3 ± 1.2 ^h^	14	41.1 ± 0.3 ^k^	19
*Vigna unguiculata*	274.2 ± 4.4 ^d,e^	27	70.8 ± 1.0 ^f^	32

* ILVs, indigenous leafy vegetables: values are means ± SD; values within a column not sharing a common superscript letter (a,b,c,d,e,f,g,h,i,j,k,l) are significantly different (Tukey ≤0.05). ^1^ RNI, recommended nutrient intakes by WHO/FAO [15]; values as (milligram) for female adults (19–50 years).

**Table 6:** Phytate (mg/100 g fresh weight) and its ratios to minerals in ILVs *.

Botanical Names	Phytate ^1^	Phytate:iron	Phytate:zinc	Phytate:calcium
*Amaranthus spinosus*	334 ± 61 ^b^	1.43	40.35	0.07
*Amaranthus* spp.	739 ± 74 ^a^	1.04	35.71	0.05
*Bidens pilosa*	119 ± 21 ^c^	3.06	13.71	0.04
*Cleome gynandra*	120 ± 6 ^c^	0.26	25.84	0.03
*Cleome hirta*	55 ± 16 ^c^	0.08	11.84	0.01
*Corchorus trilocularis*	158 ± 3 ^c^	0.31	34.02	0.06
*Ceratotheca sesamoides*	57 ± 28 ^c^	0.14	8.43	0.01
*Ipomoea obscura*	68 ± 18 ^c^	0.29	17.73	0.04
*Ipomoea pandurata*	104 ± 27 ^c^	0.86	27.84	0.04
*Justicia heterocarpa*	103 ± 8 ^c^	0.83	13.60	0.01
*Sesamum angustifolium*	146 ± 30 ^c^	5.16	20.09	0.05
*Sonchus luxurians*	44 ± 11 ^c^	0.09	10.90	0.02
*Vigna unguiculata*	109 ± 26 ^c^	1.81	26.33	0.02

* ILVs, indigenous leafy vegetables: ^1^ values are means ± SD; values within a column not sharing a common superscript letter (a,b,c) are significantly different (Tukey ≤ 0.05); Critical values of molar ratios predicting the inhibitory effect of phytate on Fe, Zn, and Ca: phytate:iron >1, phytate:zinc >15, phytate:calcium >0.24 [46].

## Appendix A

**Table A1:** Weights of ILVs before and after freeze drying in grams *.

Botanical Names	Fresh Weight ^1^ (gram)	Dried Weight ^2^ (gram)	Moisture Loss ^3^ (gram)	Moisture Loss ^4^ (%)
*Amaranthus spinosus*	10.048	1.531	8.517	84.76
*Amaranthus* spp.	38.660	15.99	22.67	58.64
*Bidens pilosa*	10.216	1.591	8.625	84.43
*Cleome gynandra*	45.380	6.510	38.87	85.65
*Cleome hirta*	68.430	9.170	59.26	86.60
*Corchorus trilocularis*	12.744	2.257	10.487	82.29
*Ceratotheca sesamoides*	205.99	47.47	158.52	76.96
*Ipomoea obscura*	86.380	7.480	78.900	91.34
*Ipomoea pandurata*	166.59	19.50	147.09	88.29
*Justicia heterocarpa*	12.937	2.479	10.458	80.84
*Sesamum angustifolium*	9.179	2.298	6.881	74.96
*Sonchus luxurians*	11.903	1.453	10.450	87.79
*Vigna unguiculata*	207.39	36.21	171.18	82.54

* ILVs, indigenous leafy vegetables: ^1^ Fresh weight = weight before freeze drying; ^2^ Dried weight = weight after freeze drying; ^3^ Moisture loss = fresh weight − dried weight; ^4^ Moisture loss = Moisture loss /Fresh weight × 100.
